# Parkinson’s Disease EMG Data Augmentation and Simulation with DCGANs and Style Transfer

**DOI:** 10.3390/s20092605

**Published:** 2020-05-03

**Authors:** Rafael Anicet Zanini, Esther Luna Colombini

**Affiliations:** Laboratory of Robotics and Cognitive Science (LaRoCS), Universidade Estadual de Campinas (UNICAMP), Campinas SP 13083-852, Brazil; esther@ic.unicamp.br

**Keywords:** Parkinson’s disease, sEMG, DCGAN, style transfer, signal processing

## Abstract

This paper proposes two new data augmentation approaches based on Deep Convolutional Generative Adversarial Networks (DCGANs) and Style Transfer for augmenting Parkinson’s Disease (PD) electromyography (EMG) signals. The experimental results indicate that the proposed models can adapt to different frequencies and amplitudes of tremor, simulating each patient’s tremor patterns and extending them to different sets of movement protocols. Therefore, one could use these models for extending the existing patient dataset and generating tremor simulations for validating treatment approaches on different movement scenarios.

## 1. Introduction

As one of the most common neurodegenerative diseases that affects approximately 10 million people around the world [1], Parkinson’s Disease (PD) has been studied and investigated from different manners and perspectives, in order to minimize the disease’s symptoms and impairments to patients.

Many studies around rest and action tremors have been conducted, whereas surface electromyography (sEMG) stands out as one of the most common ways to measure muscle response to voluntary or involuntary stimulation, being widely used as main input and feedback signal for artificial stimulation devices [2,3,4]. EMG is widely used clinically for the diagnosis of neurological and muscular pathology [5], and has recently been used for several human–machine interface applications, such as controlling computer interfaces, navigation through virtual reality environments, controlling robots, drones, and other interesting applications [6].

However, acquiring such datasets from patients is a complicated and sometimes painful task. Most patients that experience unpleasant effects during such experiments, such as tiredness, fatigue [7], and a wide range of movements, are usually not possible due to the patient’s movement limitation and impairment due to the disease.

Therefore, collecting, processing, and using recorded EMG signals for analysis is quite a challenging approach, due to data scarcity and lack of dataset variation. Data augmentation is a promising alternative approach for extending existing datasets, which could allow further research and analysis.

In this work, we propose two new data augmentation approaches based on Deep Convolutional Generative Adversarial Networks (DCGANs) and Style Transfer for augmenting Parkinson’s disease electromyography (EMG) signals with the use of two distinct EMG databases. To the best of our knowledge, this work proposes the first methods for EMG augmentation based on real patient data.

## 2. Related Work

Biological signal simulation can be used for many applications. However, generating realistic models requires a profound understanding of the simulated signal patterns and morphology [2]. Since PD’s tremor pattern is caused by a pathology with different intensity and manner for each patient, it is quite challenging to create such a generic mathematical model that can effectively produce an artificial signal similar to the real one.

Hamilton-Wright has presented a Physiologically Based Simulation for needle EMG [8], which simulates how individual motor units (MUs) are triggered, and how the relationships are between quantitative features of EMG signals and muscle structure and activation. Ahad [5] has successfully simulated EMG signals, considering different parameters that affect motor unit triggers, such as Muscle excitation, recruitment range, firing rate, and other parameters. However, his work simulates the effect of a simple contraction force on a specific muscle (tibialis anterior), for which all the required parameters have been studied and are well known. It does not simulate different movements of the muscle or the effect of a repetitive pattern, such as those combining contractions and relaxations existing on an involuntary tremor pattern.

Guerrero et al. [9] propose a complete mathematical package implemented in R for pre-processing and simulating EMG signals. Their simulation method is based on a simple heteroscedastic model-based approach, which generates a generic EMG signal. However, the EMG it created based on specific parameters such as EMG base frequency, signal length, active window size, signal mean value, standard deviation, sampling rate, and a custom shape factor. This method requires that the simulated signal is deeply understood, must be very regular, and always follows the same pattern. Such an approach cannot adapt to different movement protocols or adapt to an individual’s specific EMG tremor pattern, whose parameters are not known a priori, and present typical irregularities on frequencies and shape through time. Such adaptability is desired when trying to augment a specific patient dataset, instead of generating generic tremor patterns.

Previous work from the authors [10] has shown that it is possible to use EMG signals using neural networks to predict tremor patterns in advance. This method could enable real-time assisting devices, like Functional Electrical Stimulation (FES) devices, to operate with much more precise control over the stimulus and the patient’s tremor. However, this previous work focused on predicting a specific tremor pattern in time but did not provide a generic tremor simulator based on EMG signals. In addition, the adopted metric (RMSE), used to compare the prediction with the real signal, cannot be used when trying to generate a synthetic signal, since the shape and amplitudes might vary on time, despite keeping the signal main frequency components.

In this work, we propose two new approaches to generate surface EMG signals based on existing datasets. In our first proposed method, Neural Networks are trained to learn the specific EMG signal tremor patterns, hence being able to reproduce such tremor for each patient. The resulting model can also be employed as a feature extractor model, allowing us to further combine it with style transfer techniques for the second method. The resulting combination will enable us to generate a transformation model that simulates the tremor pattern not only on the original movement protocol but on other movements based on datasets from healthy individuals. Such extension allows us to use healthy patients datasets to investigate how PD can affect patients’ movements, on a much broader perspective than those that we can collect with real patients during measurement experiments.

## 3. Materials

### 3.1. Data Acquisition and Pre-Processing

This section describes in more detail the two EMG databases used for this work and pre-processing methods.

#### 3.1.1. Parkinson’s Disease EMG Dataset

A private research dataset from real PD patients surface electromyography (sEMG) was obtained and used with permission from the authors from previous work [11]. The dataset consists of 18 different record sets, each one with 116,000 data points (approximately 60 s), acquired from multiple sessions from five patients, one diagnosed with Essential Tremor (ET) and four diagnosed with PD according to UK Parkinson’s Disease Society Brain Bank Clinical Diagnostic Criteria [12]. All four PD patients have been diagnosed with primary PD, idiopathic usually by old age, and responsive to dopaminergic medication. All acquisition procedures were previously submitted and approved by the Plataforma Brasil ethical committee, and the patient selection was performed by neurologists from the Federal University of Sao Paulo (UNIFESP).

**Figure 1:**
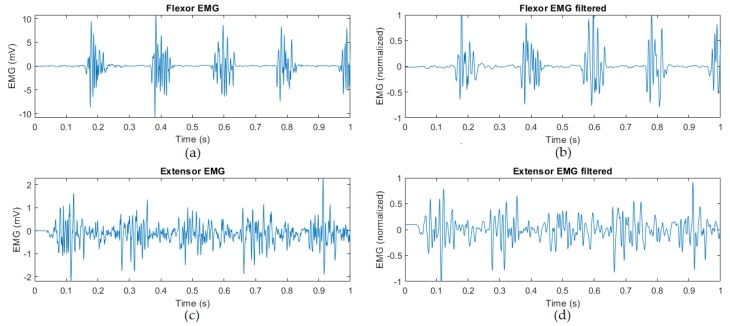
(**a**) Flexor and (**b**) Extensor EMG signals, acquired with surface EMG sensors attached to the patient’s forearm. (**c**) Flexor and (**d**) Extensor EMG signals, after 10-point moving average, re-scaling, and mean subtraction.

EMG signals were collected from wrist extensor and flexor muscles with a 2 kHz Delsys Trigno™EMG system (DELSYS INCORPORATED, Natick, MA, USA). The data collection and pre-processing methods follow the same approach from previous work [11]. Additionally, a 10-point moving average filter was applied to remove noise and high frequencies. All signals were re-scaled between –1.0 and 1.0, and have been subtracted by the signal mean to have a standard scale between different experiments and samples. The left images in Figure 1 show examples from the raw input data. The right images depict the signal after smoothing, and re-scaling processes are applied.

#### 3.1.2. NinaPro Dataset

NinaPro is an open EMG database (http://ninapro.hevs.ch/) offering different kinds of EMG readings from different sets of patients. In this work, NinaPro database two (DB2) was used [13], where data are collected from three exercises [14]:

1. Basic movements of the fingers and the wrist
2. Grasping and functional movements
3. Force patterns

Since we are interested in using consistent EMG readings from the wrist of patients, we have selected the functional movement’s experiments. We only used the readings from eight electrodes from a Delsys Trigno™Wireless EMG system (DELSYS INCORPORATED, Natick, MA, USA) (www.delsys.com) that follows a similar acquisition system like the one employed in our private PD dataset, positioned around the forearm in correspondence to the radio humeral joint [14]. The sEMG signals are also sampled at a rate of 2 kHz, which is consistent with the PD patient readings we have used before.

During the acquisition, the subjects were asked to repeat the movements with the right hand. Each movement repetition lasted five seconds and was followed by three seconds of rest. The protocol includes six repetitions of 49 different actions (plus rest) performed by 40 intact subjects [15].

**Figure 2:**
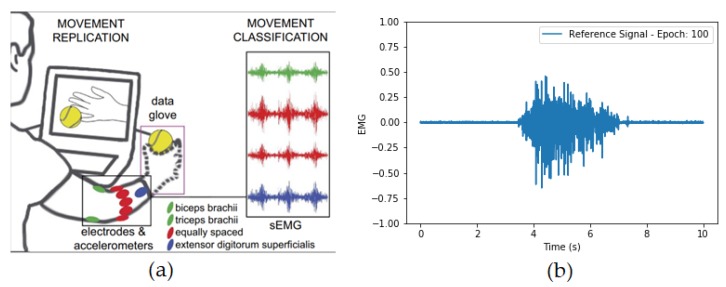
(**a**) Figure extracted from [13] showing the sensor disposition around the forearm. (**b**) Reference signal from NinaPro database 2 for one of eight EMG channels from the Delsys Trigno acquisition system after pre-processing steps.

In this work, the data from the same type of exercises (wrist flexion or extension) were combined with the pause periods to create one segment per each stimulus. The signals were also re-scaled between –1.0 and 1.0 and were subtracted by the mean. For this data, no moving average filter was applied, since we wanted to keep the original frequencies and characteristics of the content unchanged, to make sure it would be possible to compare the resulting signal to other existing models. Figure 2 shows an example of such a signal for one individual performing a wrist extension. In this work, we have selected the first eight signals, since they are the ones connected to the Delsys Trigno wireless sensors and are similar to our PD EMG dataset.

#### 3.1.3. Programming Language and Libraries

The main programming language used to implement the proposed models was Python 3. All existing implementations used for DCGAN and Style transfer were based on existing repositories written in Python. The main libraries used are open-source, available libraries:

- Tensorflow: used as the main back-end for tensor calculation
- Keras: used as an abstraction layer for creating NN models
- Jupyter Notebook: used as main python IDE for structuring code and scripts
- Pandas: used for the dataset and CSV processing
- NumPy: a scientific computing library which provides an efficient matrix and array calculations
- Matplotlib: a plotting library
- distance: library for calculating DTW distance
- fastdtw: library for calculating a faster implementation for DTW distance.

All proposed models and code are available on the following GitHub repository: https://github.com/larocs/EMG-GAN.

#### 3.1.4. Computational Resources

For this work, most of the training and evaluation of models were done on an HP ZBook 15 G3 notebook (HP Inc., Palo Alto, CA, USA), with an Intel Core i7 processor (Intel Corporation, Santa Clara, CA, USA), 32 GB of random access memory (RAM), and an NVIDIA Quadro M2000M GPU (NVIDIA CORPORATE, Santa Clara, CA, USA) with 4 GB of RAM. When additional computational power was needed, ml.p3.2xlarge instances from AWS SageMaker Notebooks (Amazon Web Services, Inc., Seattle, WA, USA) were used, which are equipped with an NVIDIA Tesla V100 (NVIDIA CORPORATE, Santa Clara, CA, USA) instance with 16 GB RAM to speed up the training process.

## 4. Proposed Methods

**Figure 3:**
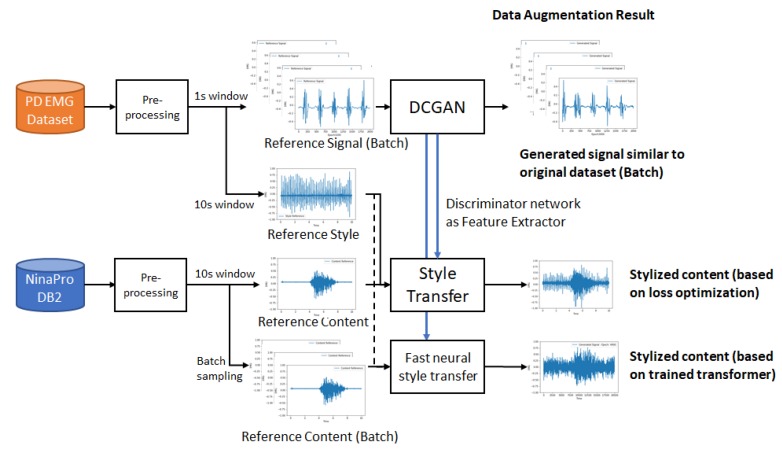
Proposed flow for the experimental setup.

This work proposed two methods for EMG data augmentation. On the first one, based on DCGANs, we train a generator that is capable of simulating each patient’s EMG tremor pattern and its correlated discriminator. In the second, based on neural style transfer and the trained discriminator from the previous method, we apply the style from a PD patient on a set of healthy patient EMG signals, simulating the expected tremor behavior on a different set of movements. We can also use the same inputs to train a Fast Neural Style Transfer transformer network to use it as a fast transformation method. Figure 3 presents a simplified diagram of the proposed methods.

### 4.1. EMG Signal Generation with DCGANs

Firstly introduced in 2014 [16], a GAN is a machine learning architecture that consists basically of two networks: a generator and a discriminator. The generator produces data with the same dimensions as those of training data, based on some latent space given as input. The discriminator tries to distinguish the input that came from the training data from the generated data. Both networks are trained through common steps, while the generator gradually gains the ability to create data that are similar to the training data, the discriminator keeps trying to force the generator to improve by providing a better classification between fake and real data.

There have been many variations and enhancements to this architecture so far (as listed on [17]) trying to optimize different aspects of such a model, like convergence, time to train, or the variety of generated samples. The DCGAN variation was introduced by [18] as an extension of the GAN architecture, where deep convolutional neural networks are employed for both the generator and discriminator models. The authors also added some general recommendations for applying DCGANs, which is intended to create a faster and more stable convergence of both generator and discriminator models, such as:

1. Replacing pooling layers with stridden convolutions for the discriminator and fractional-strided convolutions for the generator.
2. Using batch norm in both the generator and the discriminator.
3. Removing fully connected hidden layers for deeper architectures.
4. Using ReLU activation in the generator for all layers except for the output, which uses tanh.
5. Using LeakyReLU activation in the discriminator for all layers.

Based on DCGAN architecture, further developments have been made allowing GANs to be widely utilized for producing multiple images, with current developments allowing the creation of amazing high-resolution images [19].

However, despite their current success and results that focused on image generation, DCGANs have been less explored on time series and biological applications, where we shift to a multi-variable 1D context with intricate patterns varying through time. Yang et al. [20] and Engel et al. [21] present the usage of GANs for generating sound waves and music, while [22] and [23] present the usage of GANs for generating EEG and ECG signals, respectively. These works show the feasibility of using such architectures for bio-signal generation, using Wasserstein GAN (WGAN) architecture on [22] and bidirectional long short-term memory (BiLSTM) networks on [23]. Both works focused on also generating one single channel from the original signals, with a lower quantity of data points and complexity of patterns.

**Figure 4:**
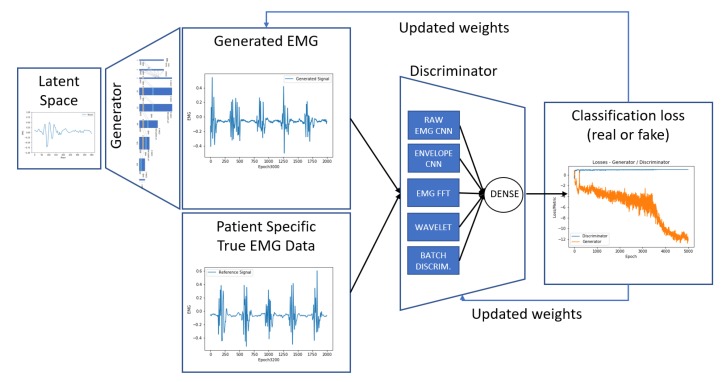
The proposed DCGAN architecture. Based on 400 points sampled randomly from the real dataset, the generator generates 2000 points that simulate the behavior of real patient tremor. The discriminator tries to distinguish the real data from the generated data, and both networks are updated based on the combined losses from the classification.

In this work, the DCGAN implementation available in [24] was used as a baseline, and the generator and discriminator networks were extended to capture more relevant features from our EMG datasets. Figure 4 shows the best architecture achieved for the proposed system. The resulting assessment was based on the proposed metrics defined in Section 4.3 and on visual perception of the signal similarity. For every change in model parameters or architecture, models were re-trained from scratch with the same dataset to compare results.

Different architectures for the discriminator and generator were evaluated, including LSTM on both models. However, due to the complexity of the tremor signal and high length of the generated signal, those strategies took too long to train and have not achieved good results.

Typically, while creating GANs, the generator is of primary interest—the discriminator is an adaptive loss function that gets discarded once the generator was trained. However, as we present in this paper, the trained discriminator can also be used as a feature extractor that can be applied in combination with other techniques, such as style transfer.

The generator is denoted as *G* (or $G_{\theta }$ when considering the parameters), and the discriminator is expressed as *D* (or $D_{r}$ when considering the parameters). A zero-sum game between the generator *G* and the discriminator *D* is performed incrementally, according to the original GAN idea to reach the Nash equilibrium point [16]:

(1) $$\min_{G}\max_{D}V\left(D,G\right)=E_{x∼p_{data}\left(x\right)}\left[logD(x)\right]+E_{z∼p_{z}\left(z\right)}\left[log(1D(G(z)))\right]$$

#### 4.1.1. Generator Model

A typical DCGAN generator proposed by Radford et al. [18] tries to generate 3-channel RGB images from a latent space *z*, given by a random sample of numbers with length nz. The generator combines several up-sampling and 2D convolutional layers, finally generating a 3-channel RGB output with the same dimensions as the original training dataset.

**Figure 5:**
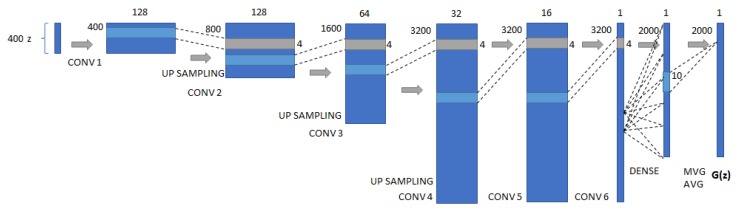
Our best proposed DCGAN Generator (*G*) model adaptation to 1D convolutions, taking 400 random samples from the training dataset and applying a sequence of convolutions, up-sampling, and a final dense and moving average layers for improving the generator’s performance while generating EMG signals.

Our best generator model consists of a deep convolution network that takes 400 point samples (0.2 s) from the original sample and tries to generate a new dataset with 2000 points (1 s). It includes on the end of the deep convolutional layers a moving average function that tries to smooth the generated signal so it can be compared to the filtered EMG input signal. We have evaluated several different parameters (such as the number of filters, layers, activation functions, and other settings) and reached a fine-tuned architecture according to the parameters shown in the experimental results. Figure 5 presents our custom implementation, adapting the convolutional layers for 1D convolutions and including a dense layer and moving average at the end of the generator pipeline.

#### 4.1.2. Discriminator Model

**Figure 6:**
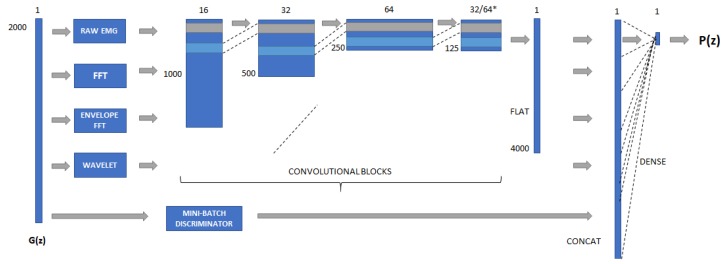
Proposed architecture for the DCGAN discriminator. Four features are extracted based on the generated signal (G(z)) and are passed through four convolutional layers. * The last convolution is adapted between 32 or 64 filters according to the different features. All filters are flattened and merged, and finally put through a dense layer with sigmoid activation for the output P(z), determining the probability of a sample being a fake or real one. The mini-batch discriminator is also merged to the convolutional filters before the last dense layer is applied.

Our best discriminator model (*D*) consists of a deep convolution network that takes a batch of 100 randomly distributed samples with 2000 sequential points and tries to distinguish if they come from the training dataset or the generator. For such a task, we have combined parallel deep convolutional pipelines where each one generates extended features based on the input vectors. The pipeline combines four convolutional stacks, as presented in Figure 6:

##### Convolutional Filters on Raw Signal

This pipeline applies four convolutional layers, each one consisting of a combination of the layers Conv1D + BatchNorm + ReLU + Dropout. The convolutional layers are applied to the raw EMG data to project the signal into 32 filters. The result is combined with other filters with a simple concatenation.

##### Convolutional Filters on FFT

The same convolutional pipeline is applied to the FFT of the raw signal, which is obtained with a custom lambda function on the input tensor. The pipeline provides a condensed representation of the frequency domain for the signal, highlighting the tremor frequencies typical for each patient.

##### Convolutional Filters on an EMG Envelope Signal

**Figure 7:**
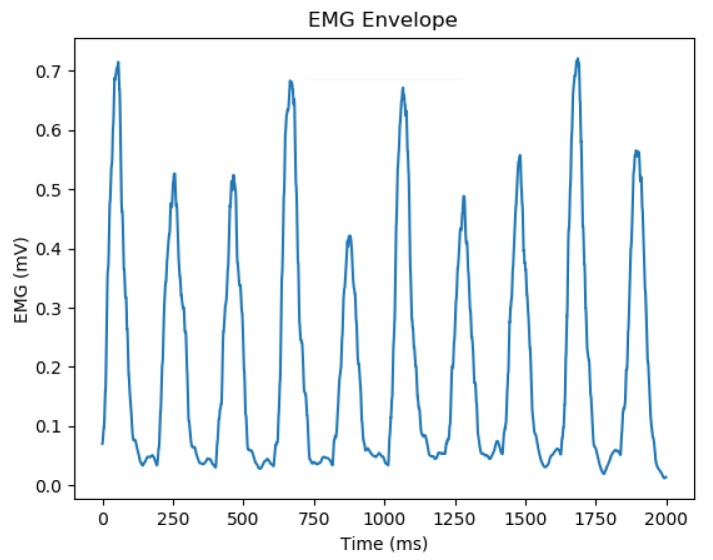
EMG envelope utilized on the DCGAN discriminator pipeline.

The same convolutional pipeline is applied to the EMG envelope, which is obtained by getting the absolute value of the EMG signal and using a moving average window with 100 points. The resulting EMG peaks are known as EMG envelopes and provide an easier detection of tremor peaks, as shown in Figure 7.

##### Convolutional Filters on Wavelet Expansion

The same convolutional pipeline is applied to a 2-level discrete wavelet transformation (DWT) of the signal, using the Daubechies wavelet db7 as wavelet mother. DWT uses a high-pass filter to obtain high-frequency components and a low-pass filter to capture low-frequency components. According to [25], this family of wavelet functions can properly extract essential features from sEMG signals, which could be successfully used for movement classification.

##### Mini-Batch Discrimination

**Figure 8:**
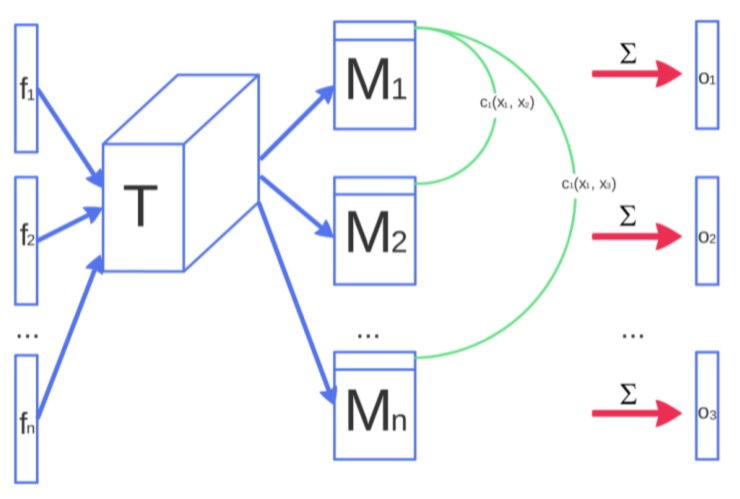
Mini-batch discriminator architecture. Features $f(x_{i})$ from sample $x_{i}$ are multiplied through a tensor **T**, generating a matrix **$M_{i}$** for every sample. Cross-sample distance is computed by the L1-distance between the rows of **$M_{i}$** across samples i∈1,2,…,n and apply a negative exponential. The output **$o(x_{i})$** for this mini-batch layer for a sample **$x_{i}$** is the sum of the $cb(x_{i},x_{j})$’s to all other samples—Reproduced with permission from [26].

The concept of mini-batch discrimination was introduced by [26] as a way to solve the issue with GANs and mode collapse. Mode collapse is when a generator learns how to generate a sample that fools the discriminator, but only for one particular case. The mini-batch discriminator adds a similarity function to the discriminator, so it can compare multiple instances of the generated data and make sure they differ from each other as a regular dataset would. This approach assures that the generator can generate multiple diverse examples that match the criteria from the discriminator. Figure 8 depicts an example of a mini-batch discriminator model.

#### 4.1.3. Evaluated Architectures

All models were implemented with the Keras framework using Tensorflow, with a default Adam optimizer with a learning rate of 0.002 and a default training period over 5000 epochs. For improving generalization of the models, dropout layers were introduced between convolutional layers, and the results presented consider models trained for only one individual for the sake of comparison.

Different activation functions were also evaluated, and the best results were achieved with the given recommendation from [18], using rectified linear units (ReLU) on generator hidden layers and LeakyReLU on all discriminator layers, and hyperbolic tangent (tanh) for the output layer from the generator.

For the DCGAN architecture, different architectures were evaluated for the generator and discriminator models. For this work, we highlight six different models, each one introducing an important improvement from the previous model:

1. 3CNN-NOISE: this model uses the generator model as described in Figure 5, with the difference of not having the last moving average layer. It uses only three of the proposed convolution filters: the raw EMG, the FFT, and the envelope FFT. We have initially evaluated this model with a typical set of 100 random points as latent space (*z*). However, we recognized that, due to the randomness of the input data, it was challenging for the generator to create a consistent and smooth time series, with the input data varying so much.
2. 3CNN: this model follows the exact same architecture as the previous model, with the distinction that it uses a 400 point sample from the reference signal. This increase in the latent space dimension (from 100 to 400) and the use of a coherent time-series signal allowed the resulting signal to be much smoother and closer to the reference signal. As we can see from the results in Table 1, the DTW and FFT metrics are much closer to this model than the previous one, and the only difference is the latent space used for the generator.
3. WAVELET: this model uses the same generator from previous models and a 2-level wavelet decomposition as a feature for the convolutional filters on the discriminator. This model was created to evaluate the effectiveness of wavelet decomposition as a feature extractor for the EMG signal. Results show that even with just 2 level decomposition, the signal is quite close to the expected tremor pattern, and therefore we decided to include such feature on the next models.
4. 4CNN: this model uses the same generator from previous models, and employs the four proposed feature pipelines. This model presented the best results. However, the generated samples are very similar to each other, indicating a case of mode collapse.
5. 4CNN-MBD: this model uses the same generator from previous model (4CNN), but includes one additional pipeline for a mini-batch discriminator (MBD) block. We can see that the resulting signal is not as smooth as the previous one, but this model generates a whole batch of different signals that are quite similar to the real samples. For creating a multi-purpose generator, the possibility of generating a whole set of distinct samples is relevant. In this sense, this model is an improvement of the previous one, although the quality is not as good.
6. 4CNN-MBD-MA: this model uses the same discriminator from the previous model (4CNN-MBD), with an additional layer of 10-point moving average (MA) at the end of the generator model. This small change enabled the model to avoid mode collapse (as we kept the mini-batch discriminator) and improved the generated samples’ quality, reducing their DTW distance and FFT MSE to the reference signals. This is the reference model (EMG-GAN) considered for further analysis.

### 4.2. EMG Generation Combining Two Signals with Style Transfer

Style Transfer (ST) was introduced in 2015 on the computer vision domain as a technique that allows us to recompose the content of an image in the style of another [27]. It has been widely used for social apps that allow the addition or removal of facial features (like aging, beards, glasses, etc.), or stylize a picture according to a famous artist, such as VanGogh, DaVinci, or Kandinsky.

The neural style algorithm introduced in [27] uses pre-trained models (VGG16) [28] as feature extractors for images, using learned features to define the semantic loss terms ($L_{cont}\left(c,x\right)$ and $L_{sty}\left(s,x\right)$) and then uses these terms to pose the optimization problem for style transfer. The output image is synthesized by an optimizer that tries to minimize both loss functions, finding an image that simultaneously matches the content representation and the style representation. In this work, we have adapted the implementation to 1D time-series data, making adjustments on the proposed content loss and style loss functions.

One disadvantage of such an approach for optimization relies on obtaining a stylized signal based on a specific input content signal. Indeed, we need to run the whole optimization process with both signals as input, the content, and the style. This approach requires a slow iterative optimization process. Therefore, it is not suited for real-time style transfer, or for a more generalized model that can stylize any given input signal.

#### 4.2.1. Content Loss

The content loss ($L_{cont}$) is based on the mean squared error (MSE) of a given content feature layer ($F^{l}$) between the content signal ($F_{cs}^{l}$) and the generated signal ($F_{gs}^{l}$). The feature layer can be used with the raw data or any other layer from a model used as a feature extractor. In our case, we have used the first convolutional layer from the raw EMG convolutional stack used within our discriminator. The deeper the convolutional layer chosen, the more abstract are the filters, and therefore the less similar the generated signal is to the content waveform. When the generated signal feature layer is identical to the one from the content, the content loss is zero.

The content loss can be expressed with the following equation:

(2) $$L_{cont}=\frac{1}{n}\sum_{i=1}^{n}{\left(F_{cs}^{l}-F_{gs}^{l}\right)}^{2}$$

During the evaluation of the model, we have identified that the competition between the content loss function ($L_{cont}$) and the style loss function ($L_{sty}$) made it impossible for the optimizer to create an output with higher amplitudes of tremor on the static part of the signals, since $L_{cont}$ tries to keep the amplitudes close to the content signal by the nature of the mean squared error (MSE). Therefore, it was necessary to add a custom “EMG content loss function” ($EL_{cont}$) that applies a mask on top of the content loss to limit its influence only on the dynamic part of the content signal. The EMG mask was configured to increase the importance of the content loss on parts of the signal whose amplitude is higher than a specific threshold value ($\epsilon_{cs}$). Then, for those parts, the difference between the content features from the content signal and the generated signal is multiplied by an amplification factor ($\alpha_{cs}$), to make sure that, for those critical points, the output will be closed to the content signal. The custom EMG content loss ($EL_{cont}$) can be defined according to the following Equation (3):

(3) $$EL_{cont}=\frac{1}{n}\sum_{i=1}^{n}|F_{cs}^{l}-F_{gs}^{l}|*\alpha_{cs},\mathrm{for}\left|F_{cs}^{l}\right|>\epsilon_{cs}$$

#### 4.2.2. Style Loss

The style loss ($L_{sty}$) proposed by [27] is based on multiple feature layers; each feature loss is calculated based on the Euclidean distance between the Gram Matrix for the generated signal and the style signal, multiplied by its specific weight ($\omega_{l}$). The Gram Matrix calculates the vector alignment between each feature by calculating the inner product between the feature map *i* and *j* in layer *l*. The Gram Matrix of a feature set can be expressed as the following:

(4) $$G_{i,j}^{l}=\sum_{k}{F_{i,k}}^{l}{F_{j,k}}^{l}$$

The loss function for style is significantly similar to our content loss, except that the Mean Squared Error for the Gram-matrices is calculated, instead of the raw tensor outputs from the layers. The overall style loss is the sum of each feature loss divided by the total number of feature layers (*N*) and channels (*M*). Thus, let *s* and *x* be the original style EMG signal and the generated EMG signal, respectively, and $A_{l}$ and $G_{l}$ their respective style representations in layer *l*. Thus, the contribution of each layer ($E_{l}$) is the following:

(5) $$E_{l}=\frac{1}{4*{N_{l}}^{2}*{M_{l}}^{2}}*\sum_{k}{\left(G_{i,j}^{l}-A_{i,j}^{l}\right)}^{2}$$

and the overall style loss ($L_{sty}$):

(6) $$L_{sty}=\sum_{l=0}^{L}\omega_{l}E_{l}$$

In this work, we replaced the VGG16 used in [27] by the trained discriminator network used for the DCGAN architecture. We took the four main discriminator convolutional stacks (raw signal, FFT, FFT over envelopes, and wavelet expansion) as the feature layers for the style loss, calculating the gram matrix for the convolutional filters for the style signal and the generated signal.

#### 4.2.3. Total Loss

Finally, the total loss ($L_{total}$) is the sum of the style loss ($L_{sty}$) and the custom EMG content loss ($EL_{cont}$) weighted by their respective weights, ($\omega_{sty}$) and ($\omega_{cont}$). We have evaluated different weights effect into the generated style transfer signal:

(7) $$L_{total}=\omega_{sty}*L_{sty}+\omega_{cont}*EL_{cont}$$

#### 4.2.4. EMG Transformation with Fast Neural Style Transfer

Fast neural style transfer [29] is an enhancement of the Style Transfer architecture that introduces the concept of a transformer network. It is explicitly trained to learn how to translate the content image to a stylized image with a feed-forward network, making the style transfer much faster and easier to apply on input images. It also allows its extension to videos and real-time conversion of frames.

In this work, we used the concept of fast neural style transfer to train a transformer network. This network receives an input EMG signal from a healthy individual—performing some functional actions (like wrist flexion/extension, grasping, pointing index fingers, and others)—and applies a transformation based on a PD patient EMG signal to simulate how the signal would look like if performed by a PD patient. The transformer network is trained based on a set of content examples (healthy individuals database coming from NinaPro) and the style (EMG signals from our private PD patient dataset). For calculating the losses between content, style, and transformed signals, we use the pre-trained discriminator from the DCGAN architecture as a feature extractor—thus allowing the transformer network to learn the individual patterns of each patient, according to the trained discriminator and generator.

This approach allows us to extend the usage of the discriminator not only to generate additional data for the given protocols (resting tremor) but also to transform existing datasets based on other protocols from healthy patients into a simulated signal as they were generated/performed by the PD’s patient.

**Figure 9:**
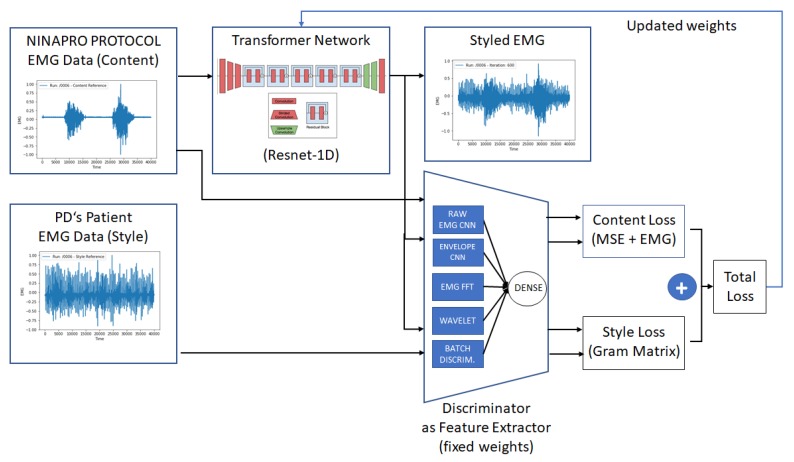
The proposed Fast Neural Style Transfer architecture. Based on sample data from NinaPro database and a PD’s patient EMG data, we train a transformer network that is capable of applying the tremor pattern on any input EMG signal. The discriminator trained during the DCGAN steps was used as a feature extractor for the Style Loss calculation, while the Content Loss was calculated based on MSE from the content signal plus a custom-designed loss for applying penalties where the EMG content signal has higher amplitudes.

The implementation was based on a Keras fast neural style transfer implementation https://github.com/misgod/fast-neural-style-keras, and we have extended the architecture to adapt the ResNet implementation for supporting 1D convolutions. Figure 9 details the proposed architecture. The transformer network was adapted from ResNet50 architecture by converting the 2D residual blocks to 1D residual blocks, including deconvolutional layers at the end, for generating an output with similar dimensions to the input.

We have also customized the implementation of the content loss and style loss, according to the architecture described in Section 4, and re-used the pre-processing routines for EMG data. For training the transformer network, we also take a randomly sampled 20,000-point window from PD EMG dataset (reference style) and a randomly sampled batch of 20,000-point windows from the NinaPro dataset.

Since the baseline implementation does not support L-BFGS (which is what the original authors used), we have used Adam optimizer. Since Adam is a first-order optimizer, this has required more hyperparameter tuning to get better results. However, creating a generic transformer network that can effectively include the style signal, and adapting it to the input content signal is a much more complex task than running an optimization function for two individual signals. Therefore, the results obtained from the style transfer are better than those from the fast neural style transfer. The same effects noticed on the first approach related to the selection of weights and features can also be extended to the fast neural style transfer.

### 4.3. Proposed Metrics

To evaluate the performance of the DCGAN and the style transfer techniques, it is important to define a group of metrics that can effectively measure the generated signal similarity to the real signals. According to Xu et al. (2018) [30], several metrics are defined for GANs, like Inception Score ([26]), Mode Score ([31]), Kernel MMD ([32]), Wasserstein distance, Fréchet Inception Distance (FID) ([33]), and many others. However, each of those metrics has benefits and disadvantages, and are usually best suited for image generators.

For evaluating the generation of time-series data, Delaney et al. (2019) [34] proposed the use of Dynamic Time Warping (DTW) and Maximum Mean Discrepancy (MMD). In this work, the authors show that both metrics can successfully evaluate the quality of the generated data, with DTW being the preferred metric since it is more robust against training instability and sensitivity to the relative amplitude between the real and synthetic data.

For style transfer, Yeh et al. (2019) [35] proposes a different set of metrics to evaluate how effectively the model transfers the style to the content based on user studies and empirical result evaluation. However, this work focuses on evaluating how good the transfer of shapes and textures between images is, which differs significantly from time-series data approaches.

In this work, we propose a set of different metrics for evaluating the result of the DCGAN model and for evaluating the style transfer.

#### 4.3.1. Fast Fourier Transform (FFT) Mean Squared Error (MSE)

Fourier analysis converts a time function into the frequency domain by decomposing the signal into sinusoidal components and the frequency domain [36]. Fourier sinusoidal components can be summed to reconstruct the time-domain waveform. Therefore, to measure the similarity between two-time series signals, one can use the mean square error (MSE) between signals FFT magnitudes. The FFT MSE was used for measuring the similarity between generated data and real data and also used to evaluate the similarity between the generated signal and the style and component signals on the style transfer step.

#### 4.3.2. Dynamic Time Warping (DTW)

**Figure 10:**
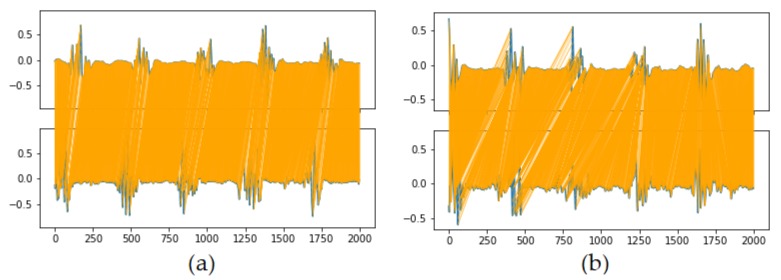
Time alignment of two time-dependent sequences using DTW. In this figure, two examples of DTW distances between real sample (upper signal) and generated sample (lower signal) for two distinct epochs: (**a**) Epoch 4800 and (**b**) Epoch 4900.

In time series analysis, DTW is one of the algorithms for measuring similarity between two temporal sequences by comparing local cost functions between both sequences. DTW has been applied to temporal sequences of video, audio, and graphics data. Recently, it has been widely used for automatic speech recognition to cope with different speaking speeds. Other applications include speaker recognition [37] and online signature recognition [38]. Figure 10 depicts a graphical representation of DTW.

Due to the large volume of data used as input and output, FastDTW was used to approximate the DTW metric as it reduces the computational time required to calculate DTW to *O(N)*, where *N* is the number of points in the series [39]. The implementation was obtained from the standard python library (https://pypi.org/project/fastdtw/).

#### 4.3.3. EMG Envelope Cross-Correlation

Cross-correlation is a measure of similarity of two series as a function of the displacement of one relative to the other. It has been commonly used for applications in pattern recognition, mainly applied to neurophysiology. The cross-correlation function is similar to applying the convolution of two functions [36]. We have used the normalized cross-correlation, which takes into account also the standard deviation and mean values of the signals, in order to have a better measure of similarity.

According to [40], cross-correlation can be useful for evaluating changes in an individual patient’s muscle activation patterns, but not for comparing EMG patterns among different individuals. Therefore, it can be an important measure for evaluating the distance between real data and fake data.

Since the shape and values of tremor peaks on EMG might vary a lot from reference and generated signals, we have identified that the simple cross-correlation on raw signals would not capture the similarity between them. Therefore, we have calculated the normalized cross-correlation between the EMG envelopes (with a 100-point moving average on absolute values—see Figure 7), in order to check if generated signals correctly captured tremor peaks.

#### 4.3.4. Style Transfer Metrics

According to Yeh et al. (2019) [35], style transfer methods are currently evaluated mostly by visual inspection on a small set of different styles and content image pairs. Such an approach could also be considered for 1D style transfer by visually inspecting the shape of resulting signals. Aiming at a more quantitative analysis over style transfer, Yeh et al. (2019) also introduces two metrics: effectiveness (E), which measures whether transferred images have the desired style; and the coherence (C), which measures the extent to which the original image’s content is preserved after the style transfer.

Typically, such metrics can be linked to the content loss and style loss functions, when using neural style transfer approach. However, experience shows that generated samples with the same values for style and content loss might show completely different qualitative results when visually inspected. Since proposed metrics such as effectiveness and coherence require user studies for evaluating the quality of the style transfer, and such studies seem unpractical for time series data, we had to propose a different approach based on the two metrics used for the DCGAN generation.

According to [41], it is possible to calculate the DTW of a multi-dimensional time series by calculating first the cross-distance matrix between all dimensions and later applying the DTW distance calculation over the matrix. In this work, we want to evaluate the DTW distance from the generated output from the style transfer process concerning the two original signals, the content, and the style signals. Considering that the content and style signals can be completely independent of each other, we can assume that the best alignment between content and style is the warping function that minimizes the distance between both signals. Therefore, it is the warping function that produces the DTW distance.

## 5. Results

This section describes the results for EMG signal generation based on the two proposed approaches.

### 5.1. EMG Signal Generation with DCGANs

**Figure 11:**
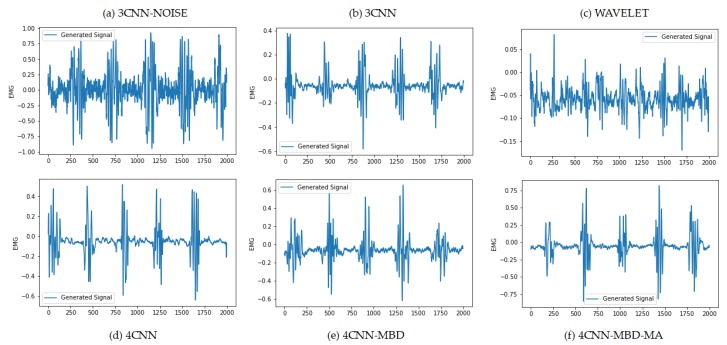
Resulting generated signals for the different evaluated models. Model details and metrics are described in Table 1 and on Section 4.1.3. (**a**) 3CNN-NOISE, resulting EMG signal is too noisy, and the tremor peaks are too wide; (**b**) 3CNN, resulting signal is smoother, but tremor shape is still far from reference signal; (**c**) WAVELET, shows promising results for capturing EMG tremor shape; (**d**) 4CNN, shows great similarity to reference, but presents mode collapse and generate very similar outputs; (**e**) 4CNN-MBD, fix the mode collapse issue, but signal is not so similar to reference; (**f**) 4CNN-MBD-MA, presents our best results, generating EMG signal similar to reference and with a lot of variation on generated samples.

The results for the different evaluated models are displayed in Table 1 and their respective generated signals are shown in Figure 11.

**Figure 12:**
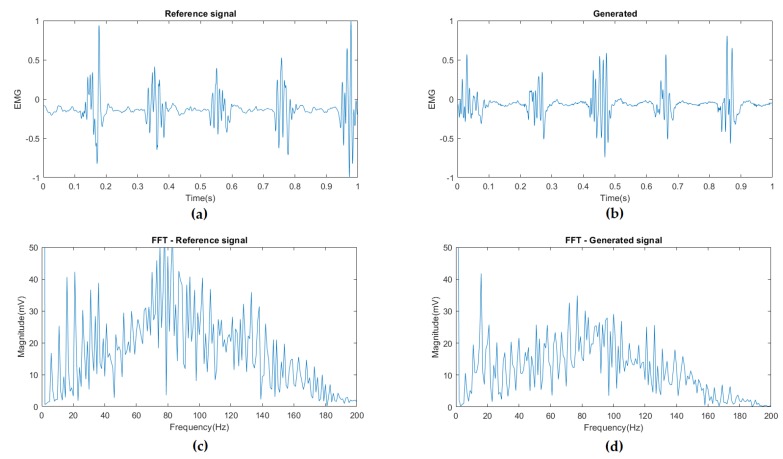
(**a**) reference input signal from PD’s EMG dataset; (**b**) generated signal based on the trained generator model after 4800 epochs; (**c**) FFT of the reference signal; (**d**) FFT of the generated signal. It is possible to see that the generated signal captures the main tremor frequencies well (around 5 Hz and its multiples, 10, 15).

**Figure 13:**
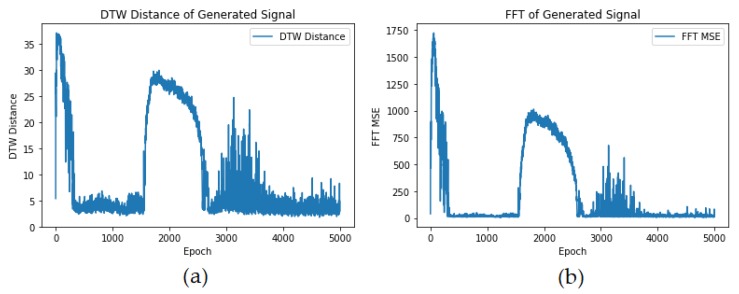
(**a**) DTW distance calculated between reference signals and generated signals along epochs; (**b**) FFT MSE calculated between reference signals and generated signals along epochs.

Figure 12 shows the comparison of real samples vs. the generated samples using the proposed EMG-GAN architecture. As we can see, the EMG signals seem quite similar, and, as Figure 13 shows, the FFT MSE and the DTW distance are very low, indicating a high similarity on the signals.

**Figure 14:**
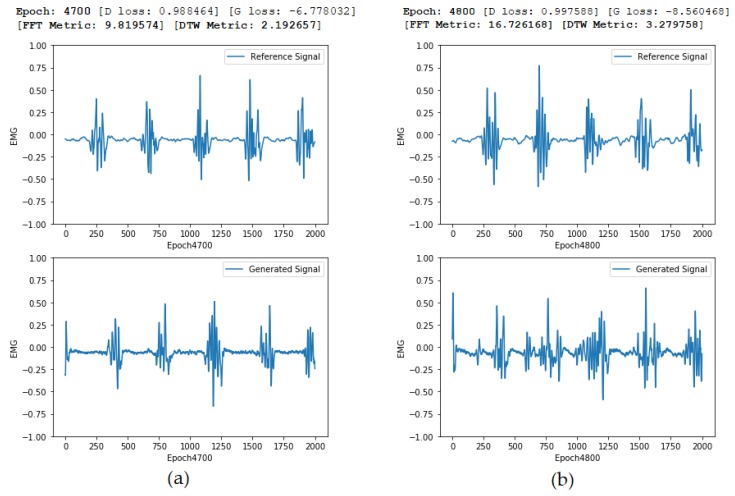
(**a**) Reference and generated signals for epoch 4700; (**b**) Reference and generated signals for epoch 4800. Even though both Generator and Discriminator losses are lower for epoch 4800, the evaluated metrics (FFT and DTW) can correctly evaluate that the first generated signal is more similar to the reference signal.

Figure 14 shows a comparison of two epochs. Although the quality of the generated signal improves over time, the visual perception of the quality of the generated signal sometimes might decrease. Both metrics DTW and FFT MSE can help to distinguish the quality of the generated signals, showing that, the lower the distance, the better the results. The cross-correlation, however, shows that the latter epoch has a higher correlation, even though the overall quality seems worse. This can happen if the similarity between the EMG tremor peaks is high, increasing the value of the correlation. This fact, isolated, does not mean that the overall quality of the generated signal is better, but it shows that the peaks are captured with higher fidelity.

**Figure 15:**
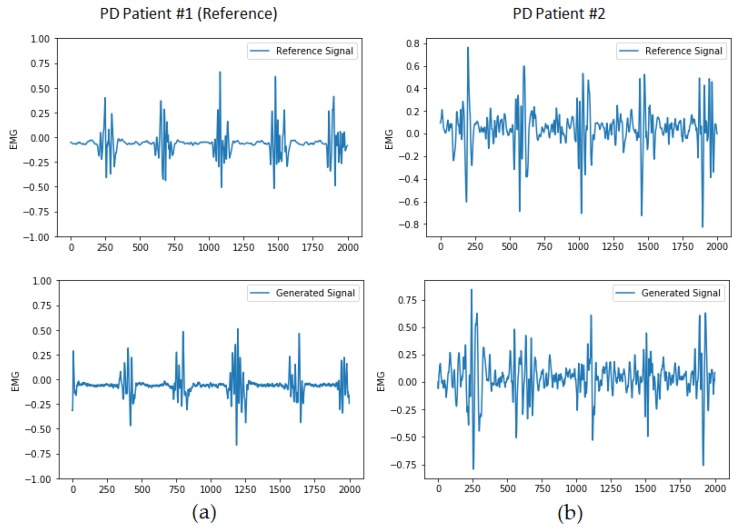
(**a**) Reference and generated signals for the PD reference patient with proposed DCGAN architecture; (**b**) Reference and generated signals for a different patient dataset.

Figure 15 shows a comparison of generated signals for two different PD’s patient datasets. Both models were trained with the same generator and discriminator architectures, with the only difference in the training dataset. As we can see, the models can effectively mimic each patient’s unique tremor pattern, showing that the model can effectively capture tremor shape, frequency, and amplitudes. Training the models with a mixed dataset from multiple patients did not give good results since the discriminator has to handle multiple patterns and does not converge.

#### Mode Collapse and Mini-Batch Discriminator

During the development of this work, it was possible to observe that the initially generated samples were quite similar, almost identical to each other, which is a shred of clear evidence that the generator is suffering from mode collapse. To prevent it from happening, we introduced the concept of mini-batch discrimination into the discriminator as an additional pipeline concatenated to the other discriminator components.

**Figure 16:**
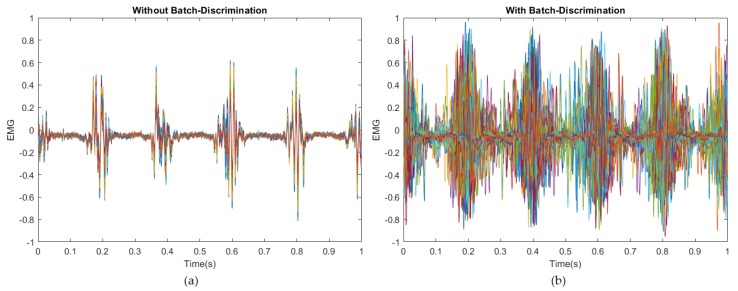
(**a**) Generated samples without mini-batch discriminator; (**b**) Generated samples with mini-batch discriminator; The results show clearly that the examples are very distinct when the Generator is trained with a mini-batch discriminator component inside the Discriminator. Without such an element, the generated samples tend to converge into a single example (mode collapse), with minimal variations on the signal shape. However, the generator takes longer to converge, and the generator loss is also affected, creating more instability in the training process.

This additional component forced the generator to produce variations on the resulting samples as it would happen into a regular random batch of real samples. However, this addition also causes some of the examples to be evaluated as fake samples by the discriminator. Figure 16 shows the results with and without the mini-batch discrimination component on the discriminator model.

### 5.2. EMG Generation Combining Two Signals with Style Transfer

**Figure 17:**
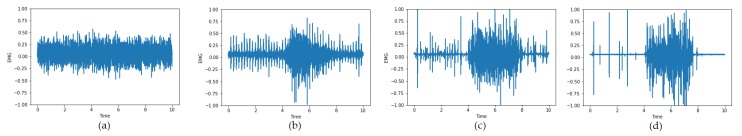
Generated outputs for different values of $\omega_{sty}$ and $\omega_{cont}$. From left to right, we gradually decrease $\omega_{sty}$ and increase $\omega_{cont}$, showing the resulting effect on the generated signal. (**a**) $\omega_{sty}=5.0$/$\omega_{cont}=0.1$; (**b**) $\omega_{sty}=4.0$/$\omega_{cont}=1.0$; (**c**) $\omega_{sty}=2.0$/$\omega_{cont}=2.0$; (**d**) $\omega_{sty}=1.0$/$\omega_{cont}=4.0$;

In this work, we took a Keras based style transfer implementation as the baseline https://keras.io/examples/neural_style_transfer/, and we re-used the pre-processing routines for EMG data and the customization of the content and style loss calculated based on features from our previous trained discriminator model. We take a 20,000 point window (equivalent to 10 s) from both PD EMG dataset (reference style) and from the NinaPro database (reference content). The optimizer used is based on Scipy library implementation of Limited-Memory Broyden–Fletcher–Goldfarb–Shanno algorithm (L-BFGS), a second-order method for optimization that, according to [27], is more suited for style transfer tasks. The output is initialized with a random vector, and we run the optimizer for 20 epochs (each epoch runs the optimization function 100 times). Figure 17 shows the comparison of results for different weights for the content weight and the style weight. It is also possible to initialize the output vector with the original content vector, which provides a faster convergence of the optimizer.

According to the selected weight values, we can have a higher level of detail from the style signal on the output signal, including not only the EMG tremor peaks but also the variations within the peaks. If we reduce the weight of the style, the peaks get higher amplitude, but the detailed EMG pattern gets lost on the style transfer. Similar behavior appears with the content weight: the lower the content weight, the lower is the amplitude and similarity of the output signal to the content signal. The higher the content weight, the more visible is the content signal within the output signal.

**Figure 18:**
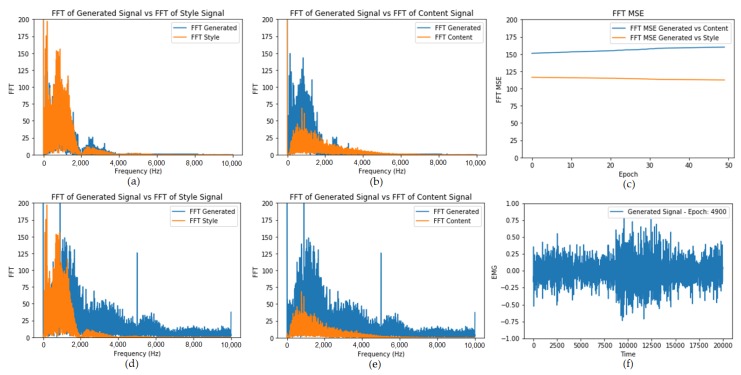
(**a**) FFT comparison between generated signal and style signal for style transfer; (**b**) FFT comparison between generated signal and content signal for style transfer. (**c**) As expected, the FFT MSE between generated signal and style decreases over time, while FFT MSE against the content increases over time, since we are using style features to drive the overall tremor frequencies on the style transfer process; (**d**) FFT comparison between generated signal and content signal for fast neural style transfer; (**e**) FFT comparison between generated signal and style signal for fast neural style transfer; (**f**) Generated output based on the fast neural transformer network.

It is also possible to change the feature layers from the discriminator model for style and content features. If we want a higher level of abstraction on the style (fewer details on the time-series), we can select the last convolutional layers as feature layers for the style loss. For the content loss, we are using the raw input signal as a feature since we want to keep the output as close as possible to the content. If we want a higher degree of abstraction also on the content, we can select deeper convolutional layers as features. In this work, we tried to keep the frequency characteristics of the style over the amplitude and keep the shape and amplitude of the content signal. Figure 18a,b present the general metrics, comparing the FFT of the generated signal against the style and content signals.

### 5.3. EMG Transformation with Fast Neural Style Transfer

Figure 18d,e shows the FFT comparison of reference samples (content and style) vs. the generated signal with the fast neural style transfer approach. As we can see, the FFT of the combined signal is much closer to the FFT of the style rather than the content FFT. However, by observing Figure 18f, we can clearly see that the shape of the content signal is still present on the resulting signal after the transformation of the content signal by the trained transformer network, showing that this method can also be used for transferring style EMG signals to content EMG signals. The results, however, are worse than those obtained with the typical style transfer based on loss optimization, as we still have a lot of undesired FFT components on the generated signal (comparison between Figure 18a,b and Figure 18d,e).

## 6. Discussion

During the development of the proposed models for DCGAN, it was possible to validate significant findings when trying to generate 1D complex bio-signals such as EMG tremor signals:

1. General recommendations from [18] are still valid and useful, such as using stridden convolutions for the discriminator, batch norm in both the generator and the discriminator, and ReLU activation in the generator for all layers except for the output.
2. While trying to replicate complex output shapes, it is essential to add different convolutional pipelines that focus on specific features from the signal. In our case, adding both FFT analysis and wavelet decomposition to the discriminator model was essential for generating better results.
3. Using 1D convolutions and adapting all blocks (like residual blocks) gave us better results than trying to use 2D representations of the time-series signals.
4. We have evaluated different architecture parameters for the Generator model, using a different number of convolutional layers, filters, and different input sizes for the latent space. It was possible to see during experiments that, with a lower and random latent space, the model takes longer to converge, and the generated signal is not as good as expected. By increasing the number of points and by introducing the sampling of the real signal as input, it was possible to generate better results.
5. Defining metrics that can effectively evaluate the performance of the generator vs. the reference signals are also important and might vary a lot depending on the features that we want to preserve from the original signals. In our case, the FFT and DWT were the best evaluation metrics for raw EMG. Normalized cross-correlation is only able to capture similarity between reference and generated signals if we apply it over the EMG envelopes, since raw EMG data vary too much over peak shapes and amplitudes.
6. Mini-batch discriminator is vital if we wish to create a generator that can create a wide range of variations on the generated signals. However, this can reduce the stability and convergence of the generator if not configured properly together with other features on the discriminator model.
7. It was possible to see that the proposed method successfully simulates given input EMG patterns, even when tremor is not well identifiable within the EMG signal, showing great potential for generalization to generate many other types of movement besides tremor patterns. The proposed architecture can be extended by Future work that can explore such potential of proposed models for other types of EMG signal applications.

For the development of the style transfer, it was essential to find a pre-trained model that can extract crucial style features from the EMG signals. In our case, re-using the discriminator trained on the previous step represented a considerable advantage, and made it possible to transfer the tremor pattern to the content signal effectively. It is also important to evaluate carefully the weights used on the style transfer since different features might affect the output result differently.

Future work could extend the results of this work by collecting new datasets from PD’s patients performing similar movements like those available on the NinaPro database (e.g., wrist extension, flexion) in order to validate that the style transfer method proposed is really an optimal approximation to real patients’ movements. This could support finding optimal weights for the content and style and also evaluate better the level of feature abstraction needed to generate a realistic sample. We could also use the proposed methods to assess the differences between primary and secondary PD patients, utilizing the discriminator models to classify both types of disease. Another possibility is to extend the DCGAN architecture for conditional input embedding (C-DCGAN), similar to the approach from [42], making it possible for the DCGAN model to adapt the generated signal based on the selected patient given as an additional parameter to the generator. To improve even further EMG signal generation, additional features from the input signal could also be explored, like using PCA decomposition or other EMG features to the discriminator model pipeline, to improve the results from the generator model.

## 7. Conclusions

This paper proposes two new data augmentation methods for EMG signal generation using DCGANs and Style Transfer, creating a reference implementation based on Python. It contributes with three main findings to its field. First, we have shown that the usage of DCGANs with domain-specific discriminator CNN pipelines can successfully simulate EMG tremor behavior, not only mimicking generic tremor patterns but patient and protocol-specific characteristics. Such achievement can support the development of new assisting treatments for reducing tremors on PD patients by extending existing datasets and reducing the necessary time for real patient experiments for capturing data. Second, we have validated that the DTW distance and FFT MSE can be defectively used as a measurement for the evaluation of EMG signal generation. Finally, by utilizing the Style Transfer approach, we were able to successfully transfer tremor patterns for different protocols and datasets, simulating patients tremor on various circumstances and protocols using existing healthy patient datasets. Such results can provide the basis for building Parkinson’s disease signal simulators, allowing patients to spend less time on data acquisition experiments, and allowing the generation of more data for supporting further assisting technology development.

## Figures and Tables

**Table 1:** Comparison table for highlighted DCGAN architectures. The lower the DTW and FFT MSE metric values, the better is the generated signal. The lower the discriminator loss, the better it is at distinguishing fake from real samples. The lower the generator loss, the better it is at generating fake samples closer to real samples. Model number 6 (4CNN-MBD-MA) shows the best overall results.

#	Model	Latent Space (z)	Disc. Loss	Gen. Loss	DTW	FFT MSE	EMG Envelope Cross-Correlation
**1.**	**3CNN-NOISE**	Rand (100)	0.000002	16.118095	405.038863	130.188808	0.373913
**2.**	**3CNN**	Sample (400)	0.005163	3.084711	132.185279	13.093062	0.205428
**3.**	**WAVELET**	Sample (400)	0.066975	2.680009	100.145536	16.564078	0.223018
**4.**	**4CNN**	Sample (400)	0.035544	7.006461	93.439412	9.675622	0.624258
**5.**	**4CNN-MBD**	Sample (400)	0.000203	10.275796	100.786512	18.916364	0.739453
**6.**	**4CNN-MBD-MA**	Sample (400)	0.004439	10.636311	98.532786	13.531477	0.791920

## References

[1] World Health Organization (2006). Neurological Disorders: Public Health Challenges.

[2] Petersen E., Rostalski P. (2019). A Comprehensive Mathematical Model of Motor Unit Pool Organization, Surface Electromyography, and Force Generation. Front. Physiol..

[3] Philipson B.J. (2009). System and Methods for Emg-Triggered Neuromuscular Electrical Stimulation. U.S. Patent.

[4] Bó A.P.L. (2010). Compensation Active de Tremblements Pathologiques des Membres supéRieurs via la Stimulation éLectrique Fonctionnelle. Ph.D. Thesis.

[5] Ahad M.A. (2019). Analysis of Simulated Electromyography (EMG) Signals Using Integrated Computer Muscle Model. Ph.D. Thesis.

[6] Morón J., DiProva T., Cochrane J.R., Ahn I.S., Lu Y. EMG-based hand gesture control system for robotics. Proceedings of the 2018 IEEE 61st International Midwest Symposium on Circuits and Systems (MWSCAS).

[7] Kostić V.S., Tomić A., Ječmenica-Lukić M. (2016). The Pathophysiology of Fatigue in Parkinson’s Disease and its Pragmatic Management. Mov. Disord. Clin. Pract..

[8] Hamilton-Wright A., Stashuk D.W. (2005). Physiologically based simulation of clinical EMG signals. IEEE Trans. Biomed. Eng..

[9] Guerrero J.A., Macías-Díaz J.E. (2019). A package for the computational analysis of complex biophysical signals. Int. J. Mod. Phys. C.

[10] Zanini R.A., Colombini E.L., de Castro M.C.F. Parkinson’s Disease EMG Signal Prediction Using Neural Networks. Proceedings of the 2019 IEEE International Conference on Systems, Man and Cybernetics (SMC).

[11] Pinheiro W.C., Bittencourt B.E., Luiz L.B., Marcello L.A., Antonio V.F., de Lira P.H.A., Stolf R.G., Castro M.C.F. Parkinson’s Disease Tremor Suppression. Proceedings of the 10th International Joint Conference on Biomedical Engineering Systems and Technologies (BIOSTEC 2017).

[12] Hughes A., Daniel S., Kilford L., Lees A. (1992). Accuracy of clinical diagnosis of idiopathic Parkinson’s disease: A clinico-pathological study of 100 cases. J. Neurol Neurosurg. Psychiatry.

[13] Atzori M., Gijsberts A., Castellini C., Caputo B., Hager A.G.M., Elsig S., Giatsidis G., Bassetto F., Müller H. (2014). Electromyography data for non-invasive naturally-controlled robotic hand prostheses. Sci. Data.

[14] Gijsberts A., Atzori M., Castellini C., Müller H., Caputo B. (2014). Movement Error Rate for Evaluation of Machine Learning Methods for sEMG-Based Hand Movement Classification. IEEE Trans. Neural Syst. Rehabiliation Eng..

[15] Atzori M., Cognolato M., Müller H. (2016). Deep Learning with Convolutional Neural Networks Applied to Electromyography Data: A Resource for the Classification of Movements for Prosthetic Hands. Front. Neurorobot..

[16] Goodfellow I.J., Pouget-Abadie J., Mirza M., Xu B., Warde-Farley D., Ozair S., Courville A.C., Bengio Y. (2014). Generative Adversarial Networks. arXiv.

[17] Lucic M., Kurach K., Michalski M., Gelly S., Bousquet O., Bengio S., Wallach H., Larochelle H., Grauman K., Cesa-Bianchi N., Garnett R. (2018). Are GANs Created Equal? A Large-Scale Study. Proceedings of the 32nd International Conference on Neural Information Processing Systems (NeurIPS 2018).

[18] Radford A., Metz L., Chintala S. (2015). Unsupervised Representation Learning with Deep Convolutional Generative Adversarial Networks. arXiv.

[19] Karras T., Aila T., Laine S., Lehtinen J. (2017). Progressive Growing of GANs for Improved Quality, Stability, and Variation. arXiv.

[20] Yang L.C., Chou S.Y., Yang Y.H. (2017). MidiNet: A Convolutional Generative Adversarial Network for Symbolic-Domain Music Generation. arXiv.

[21] Engel J., Agrawal K.K., Chen S., Gulrajani I., Donahue C., Roberts A. (2019). GANSynth: Adversarial Neural Audio Synthesis. arXiv.

[22] Hartmann K.G., Schirrmeister R.T., Ball T. (2018). EEG-GAN: Generative adversarial networks for electroencephalograhic (EEG) brain signals. arXiv.

[23] Zhu F., Ye F., Fu Y., Liu Q., Shen B. (2019). Electrocardiogram generation with a bidirectional LSTM-CNN generative adversarial network. Sci. Rep..

[24] Linder-Norén E. (2017). Keras-GAN. https://github.com/eriklindernoren/Keras-GAN/tree/master/dcgan.

[25] Mane S., Kambli R., Kazi F., Singh N. (2015). Hand Motion Recognition from Single Channel Surface EMG Using Wavelet & Artificial Neural Network. Procedia Comput. Sci..

[26] Salimans T., Goodfellow I.J., Zaremba W., Cheung V., Radford A., Chen X. (2016). Improved Techniques for Training GANs. arXiv.

[27] Gatys L.A., Ecker A.S., Bethge M. (2015). A Neural Algorithm of Artistic Style. arXiv.

[28] Simonyan K., Zisserman A. (2014). Very Deep Convolutional Networks for Large-Scale Image Recognition. arXiv.

[29] Johnson J., Alahi A., Fei-Fei L. (2016). Perceptual Losses for Real-Time Style Transfer and Super-Resolution. ECCV.

[30] Xu Q., Huang G., Yuan Y., Guo C., Sun Y., Wu F., Weinberger K.Q. (2018). An empirical study on evaluation metrics of generative adversarial networks. arXiv.

[31] Che T., Li Y., Jacob A.P., Bengio Y., Li W. (2016). Mode Regularized Generative Adversarial Networks. arXiv.

[32] Gretton A., Borgwardt K.M., Rasch M.J., Schölkopf B., Smola A.J. (2007). A Kernel Method for the Two-Sample-Problem. Advances in Neural Information Processing Systems 19.

[33] Heusel M., Ramsauer H., Unterthiner T., Nessler B., Klambauer G., Hochreiter S. (2017). GANs Trained by a Two Time-Scale Update Rule Converge to a Nash Equilibrium. arXiv.

[34] Delaney A.M., Brophy E., Ward T.E. (2019). Synthesis of Realistic ECG using Generative Adversarial Networks. arXiv.

[35] Yeh M.C., Tang S., Bhattad A., Zou C., Forsyth D. (2019). Improving Style Transfer with Calibrated Metrics. arXiv.

[36] Semmlow J., Griffel B. (2014). Biosignal and Medical Image Processing.

[37] Shahin I., Botros N. Speaker identification using dynamic time warping with stress compensation technique. Proceedings of the IEEE Southeastcon’98 ‘Engineering for a New Era’.

[38] Miguel-Hurtado O., Mengibar-Pozo L., Lorenz M.G., Liu-Jimenez J. On-Line Signature Verification by Dynamic Time Warping and Gaussian Mixture Models. Proceedings of the 2007 41st Annual IEEE International Carnahan Conference on Security Technology.

[39] Salvador S., Chan P. (2007). Toward Accurate Dynamic Time Warping in Linear Time and Space. Intell. Data Anal..

[40] Wren T., Do K.P., Rethlefsen S., Healy B. (2006). Cross-correlation as a method for comparing dynamic electromyography signals during gait. J. Biomech..

[41] Ten Holt G., Reinders M., Hendriks E. (2007). Multi-dimensional dynamic time warping for gesture recognition. ASCI 2007—Proceedings of the 13th Annual Conference of the Advanced School for Computing and Imaging.

[42] Fu R., Chen J., Zeng S., Zhuang Y., Sudjianto A. (2019). Time Series Simulation by Conditional Generative Adversarial Net. arXiv.

